# Next generation sequencing and *de novo* transcriptome analysis of *Costus pictus* D. Don, a non-model plant with potent anti-diabetic properties

**DOI:** 10.1186/1471-2164-13-663

**Published:** 2012-11-23

**Authors:** Ramasamy S Annadurai, Vasanthan Jayakumar, Raja C Mugasimangalam, Mohan AVSK Katta, Sanchita Anand, Sreeja Gopinathan, Santosh Prasad Sarma, Sunjay Jude Fernandes, Nandita Mullapudi, S Murugesan, Sudha Narayana Rao

**Affiliations:** 1 Research and Development Unit, Genotypic Technology Private Limited, Balaji Complex, Poojari Layout, 80 Feet Road, RMV 2nd Stage, Bangalore, Karnataka, 560094, India; 2 Currently at MTP Biology, ITC R&D Centre, Peenya Industrial Area, 1st Phase, Bangalore, Karnataka, 560 058, India; 3 Division of Bioprospecting, Institute of Forest Genetics and Tree Breeding, R.S.Puram, Coimbatore, Tamilnadu, 641 002, India

**Keywords:** RNA-Seq, Next Generation Sequencing (NGS), *de novo* Assembly, Abscisic Acid (ABA), *Costus pictus*, Diabetes mellitus, Bixin, Molecular signature, PPAR agonist, High Performance Liquid Chromatography (HPLC)

## Abstract

**Background:**

Phyto-remedies for diabetic control are popular among patients with Type II Diabetes mellitus (DM), in addition to other diabetic control measures. A number of plant species are known to possess diabetic control properties. *Costus pictus* D. Don is popularly known as “Insulin Plant” in Southern India whose leaves have been reported to increase insulin pools in blood plasma. Next Generation Sequencing is employed as a powerful tool for identifying molecular signatures in the transcriptome related to physiological functions of plant tissues. We sequenced the leaf transcriptome of *C. pictus* using Illumina reversible dye terminator sequencing technology and used combination of bioinformatics tools for identifying transcripts related to anti-diabetic properties of *C. pictus*.

**Results:**

A total of 55,006 transcripts were identified, of which 69.15% transcripts could be annotated. We identified transcripts related to pathways of bixin biosynthesis and geraniol and geranial biosynthesis as major transcripts from the class of isoprenoid secondary metabolites and validated the presence of putative norbixin methyltransferase, a precursor of Bixin. The transcripts encoding these terpenoids are known to be Peroxisome Proliferator-Activated Receptor (PPAR) agonists and anti-glycation agents. Sequential extraction and High Performance Liquid Chromatography (HPLC) confirmed the presence of bixin in *C. pictus* methanolic extracts. Another significant transcript identified in relation to anti-diabetic, anti-obesity and immuno-modulation is of Abscisic Acid biosynthetic pathway. We also report many other transcripts for the biosynthesis of antitumor, anti-oxidant and antimicrobial metabolites of *C. pictus* leaves.

**Conclusion:**

Solid molecular signatures (transcripts related to bixin, abscisic acid, and geranial and geraniol biosynthesis) for the anti-diabetic properties of *C. pictus* leaves and vital clues related to the other phytochemical functions like antitumor, anti-oxidant, immuno-modulatory, anti-microbial and anti-malarial properties through the secondary metabolite pathway annotations are reported. The data provided will be of immense help to researchers working in the treatment of DM using herbal therapies.

## Background

Diabetes mellitus (DM) is one of the most widely occurring metabolic disorders throughout the world which is characterized by chronic hyperglycemia as a result of insulin resistance or defect in insulin secretion. Defects in insulin secretion and/or action, results in increased blood glucose levels and the condition is termed as DM. Type 2 DM represents 90-95% of the cases and the individuals affected by this disorder generally have insulin resistance and a relative insulin deficiency [1]. Even though, there are several medicines available for diabetic management, they are associated with significant side effects that affect the quality of life. Herbal preparations also play a vital role in diabetic management. Various drug targets have been detailed for DM and the need for systematic evaluation of herbal therapeutics at molecular level has been urged to be included in medical practices [2]. Intense molecular studies on herbal remedies and the elucidation of their molecular mechanisms will bring out a potentially powerful anti-diabetic therapy and will be immensely beneficial to patients.

**Figure 1:**
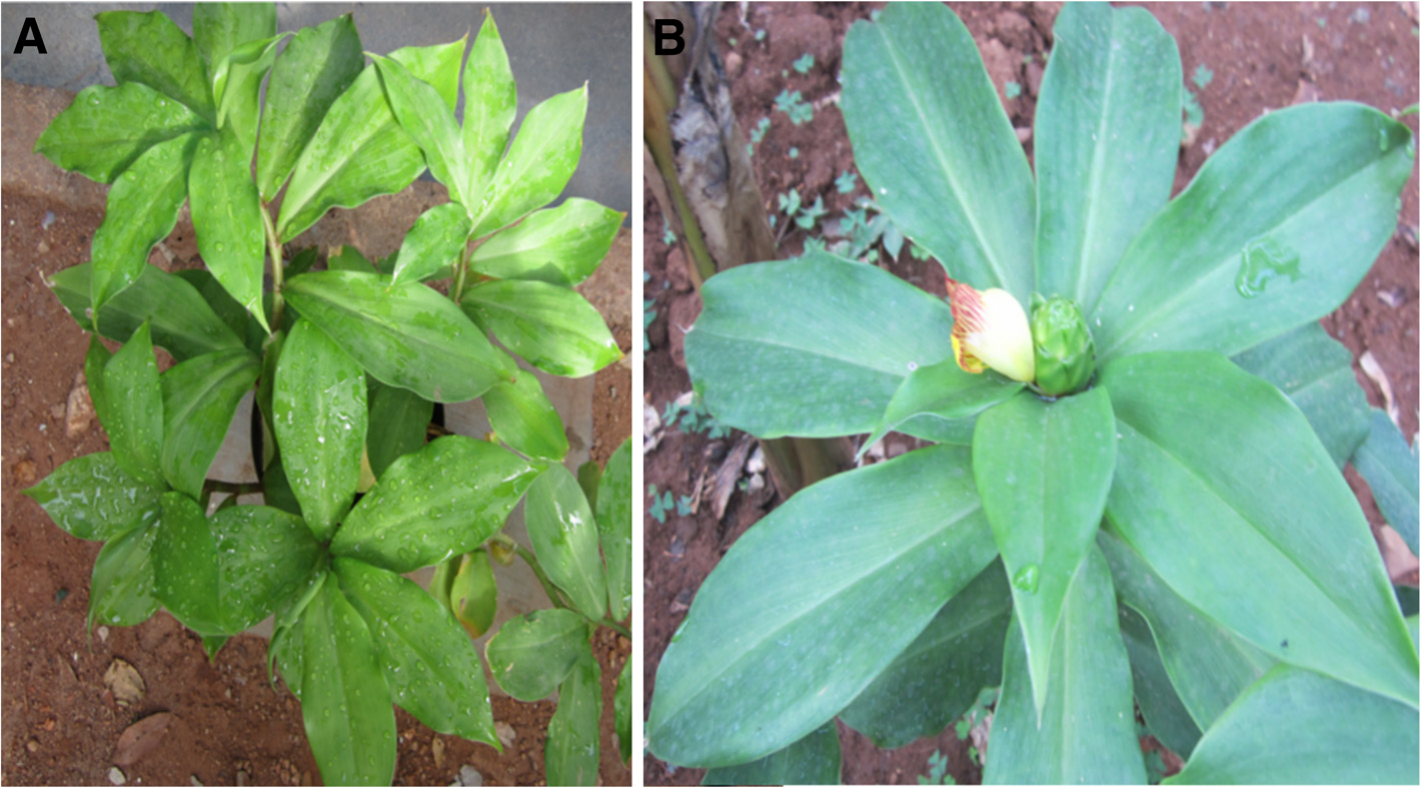
***Costus pictus D. Don *plant. A**) A young *C. pictus* growing in pot cultures **B**) A view of fully grown *C. pictus* at the flowering stage.

Many indigenous plants with different biochemical properties have been reported to possess anti-diabetic properties. *Costus pictus* D. Don (Figure 1) is one such native plant of Mexico and was introduced to India in recent years. It has gained increased popularity in recent years due to its anti-diabetic properties and is commonly called as “Insulin plant” or “Spiral Ginger” [3]. The leaves of this plant have been reported to possess anti-diabetic properties [3-9]. A patent has been filed: “Preparation process and a regenerative method and technique for prevention, treatment and glycemic control of diabetes mellitus using *Costus pictus* extract” which describes that oral supplementation of *C. pictus* (500–2000 mg) per day brings down the blood glucose levels in diabetic patients [4]; however, no commercial anti-diabetic product is available yet. Various hypotheses, on the possible mechanisms responsible for the anti-diabetic potential of the plant include i) suppression of carbohydrate hydrolysing enzymes like α-amylase and α-glucosidase [3], ii) stimulation of insulin secretory response by increasing Ca^2+^ influx through voltage gated Ca^2+^ channels [5], iii) β-amyrin as being the active and responsible component [6], and iv) PTP1B inhibition and IRβ–PI3K activation [8]. However, the exact mechanism of action of the leaves is still elusive. The anti-diabetic properties of the leaves are strongly supported by their anti-oxidant properties [9]. There have also been reports on the leaves that they work against cancer [10]. The leaves are also suggested to act as anti-bacterial and anti-glycation agents [9]. *C. pictus* is also known to be a powerful diuretic agent which is used in treatment of renal disorders [11].

Genomic analysis of *C. pictus*, a non-model medicinal plant, is limited by the small quantity of publicly available sequence data. However, the emergence of next generation sequencing has paved the way for large scale sequencing of several non-model plants which can be valuable in investigating the basis of medicinal properties of such plants. Different Next Generation Sequencing (NGS) technologies and their potential applications in plant biology including transcriptome investigations have been reviewed [12]. Strategies and tools which can be employed in transcriptome studies of non-model plants using second generation sequencing have been discussed [13]. Non-model plants that have been recently sequenced include *Daucus Cicer arietinum L.*[14], *Carota var. sativus L.*[15], *Hevea brasiliensis*[16], *Sesamum indicum L.*[17], *Ipomoea batatas*[18], *Camellia sinensis*[19], *Acacia auriculiformis*, *Acacia mangium*[20], *Cajanus cajan L*. [21], *Euphorbia fischeriana*[22], *Myrica rubra*[23], and many others are in progress. Even though many plant species are reported to be of anti-diabetic importance, the only plant that was reported to be sequenced is *Gynostemma pentaphyllum*[24]. We have undertaken an NGS based approach to sequence the *C. pictus* transcriptome in order to identify and characterize transcripts potentially contributing to the observed medicinal properties. We have confirmed the presence of a precursor to Bixin viz Putative norbixin methyltransferase. This study will aid in the understanding of the therapeutic potential of *C. pictus* and serve as a valuable resource for numerous researchers working on developing treatments for DM. Availability of this transcriptomic data in public domains will also enable genome wide comparative studies of closely related medicinal plants of anti-diabetic importance.

## Results

### Sequencing and quality control

A total of 44 million, 73 base paired-end reads (22,222,948 * 2 = 3.2Gb) were generated by the Illumina Genome Analyzer IIx Sequencer. The raw paired-end sequence data in FASTQ format is deposited in the National Centre for Biotechnology Information's (NCBI) Short Read Archive (SRA) database under the accession number SRA052634. Raw reads were subjected to quality control using SeqQC. High quality (>Q20) bases were more than 97% in both the forward and the reverse (paired-end) reads. Percentage of unresolved bases (Ns) was observed to be very minimal (0.006% in forward read and 0.149% in reverse read). The results also showed that the average Phred scaled quality score (Q score) was above 30 (>Q30) at all base positions in both the reads indicating a very high quality sequencing run. After processing adapter sequences and low quality sequences from the raw data, 41,104,416 high quality reads (~92.5% of total reads) were retained. These high quality, processed paired-end reads were used to assemble into contigs and further into transcripts.

### *De novo* assembly

**Figure 2:**
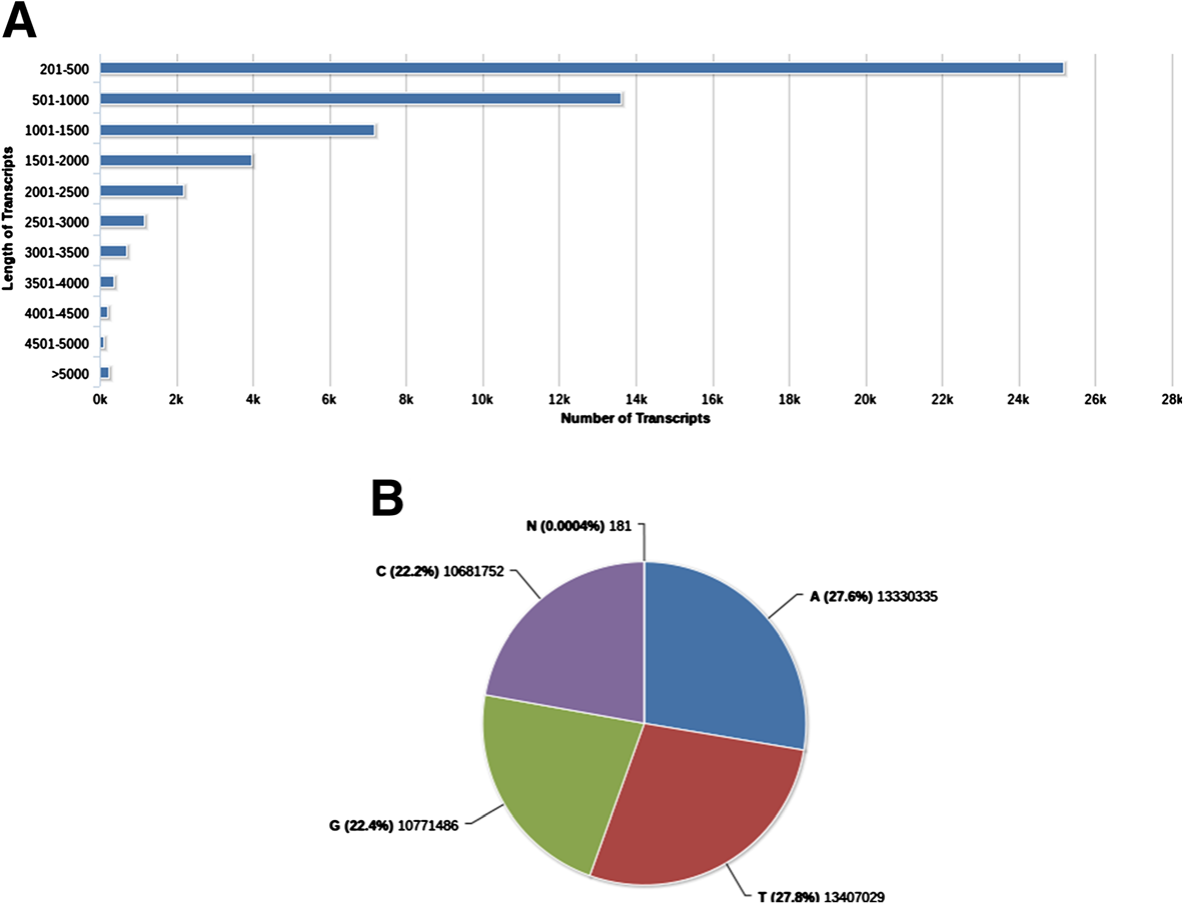
**Transcript Assembly Information. A**) Transcript Length Distribution **B**) ATGC Composition of assembled transcripts.

*De novo* assembly of the processed reads using Velvet yielded 53,416 contigs. A k-mer of 47 resulted in an optimal assembly in comparison to other k-mer assemblies based on different assembly quality parameters like N50 length, average contig length, total length of the contigs, total number of contigs, longest contig length and number of Ns. The contigs were further assembled into transcripts using the transcriptome assembly software, Oases. Transcripts which were shorter than 200 bases in length were filtered out, resulting in 55,006 transcripts. The lengths of the assembled transcripts are represented as a bar chart (Figure 2 A).

Number of unresolved bases (Ns) was found to be very minimal (181 in number). Total length of the transcripts was observed to be 48,190,783 bases (48.1 Mb) and average length of the transcripts was approximately 876 bases (Table 1). The transcripts were found to be marginally AT-rich - 55.4% (Figure 2 B).

**Table 1:** Assembly Statistics

	
Total Number of Transcripts	55,006
Maximum Transcript Length (in bases)	15,313
Minimum Transcript Length (in bases)	201
Average Transcript Length (in bases)	876.1
Total Transcripts Length (in bases)	48,190,783
Total Number of Ns	181
Transcripts > 500 b	29,835
Transcripts > 1 Kb	16,210
Transcripts > 10 Kb	9
N50 size (in bases)	1,353
GC %	44.6
AT %	55.4

N50 is a statistic widely used to assess the quality of sequence assembly. Higher the N50 value better is the assembly. The N50 in our assembly was found to be 1,353 bases, which was higher than most other plant transcriptome assemblies published, barring a few exceptions (Table 2). The assembled transcript sequences are deposited at NCBI's Transcriptome Shotgun Assembly (TSA) sequence database and are assigned GenBank accession numbers (JW214778-JW269783).

**Table 2:** Comparison of N50 values with other plant transcriptome assemblies

**Organism**	**N50 (in bases)**
*Cicer arietinum* L. [14]	1192
*Daucus carota* var. sativus L. [15]	1378
*Hevea brasiliensis*[16]	485
*Sesamum indicum L*. (3 libraries) [17]	220, 150, 180
*Ipomoea batatas*[18]	765
*Camellia sinensis*[19]	506
*Acacia auriculiformis*[20]	948
*Acacia mangium*[20]	938
*Cajanus cajan L.*[21]	~1500
*Euphorbia fischeriana*[22]	1510

### Functional annotation

Functional annotation of novel plant transcriptomes is a challenging task due to the limited availability of reference genome/gene sequences in public databases. Being a non-model plant and without much availability of reference sequences in the databases, it is challenging to predict accurate annotations for the transcripts. In order to maximise annotation percentages, six different databases (PlantCyc, UniProt: Swiss-Prot, UniProt: TrEMBL, Cluster of Orthologous Groups, Pfam and Viridiplantae mRNA), were mined. This strategy resulted in 69.15% of the transcripts being annotated. Although the TrEMBL database and the all Viridiplantae mRNA database from GenBank lacked proper annotation, they were included to increase the possibility of annotating the unknown transcripts which do not have significant similarity in well annotated databases. A six-way venn diagram was constructed to depict the sharing of transcripts annotated by the six databases (Additional file 1).

### Pathway annotation

Pathways possibly contributing to anti-diabetic, anti-oxidant, antimicrobial, anti-glycation and antitumor properties of *C. pictus* leaves reported earlier [3-10] were studied. The PlantCyc database was used to annotate 5,512 transcripts and was vital in retrieving pathways specifically from plants. Terpenoids, also called isoprenoids, are a large group of secondary metabolites which are reported to function in communication and defense, as antitumor, as anti-malarial and as anti-diabetic agents [25]. We focused on studying terpenoid pathways along with other secondary metabolite pathways (Additional file 2) to identify clues related to the medicinal properties of the plant with the help of PlantCyc annotations.

**Figure 3:**
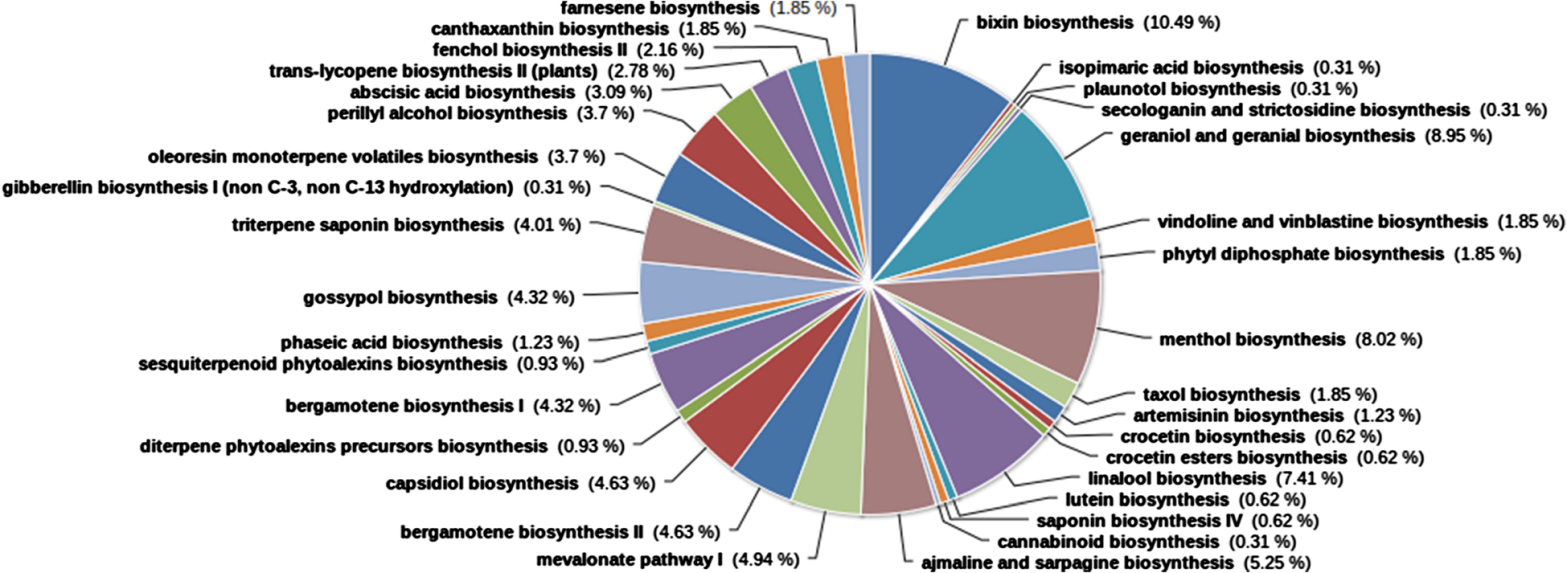
Percentage distribution of terpenoid pathway related transcripts observed from PlantCyc enzymes annotation.

The observed terpenoid pathways are represented in a pie-chart (Figure 3). A major share of the transcripts related to terpenoid pathways was noticed to be from bixin biosynthesis (10.49%) and geraniol and geraniol biosynthesis (8.95%) pathways which have been implicated with anti-diabetic functions [26,27]. Abscisic Acid (ABA) biosynthesis (3.09%) transcripts observed are also reported to have anti-diabetic functions [28,29]. Anti-oxidant properties have been reported in some of the by-products from the annotated pathways which include bixin [30], astaxanthene, canthaxanthene [31], all-trans-lycopene, lutein [32], crocetin [33], gossypol [34], saponins [35], oleoresin [36] and this correlates with the strong anti-oxidant properties of *C. pictus*. Transcripts corresponding to Menthol biosynthetic pathway were also found to occur predominantly (8.02%); the end-product menthol might contribute to the antitumor proeprties [37]. The other by-products from the annotated pathways which could potentially render the antitumor properties include taxol [38], all-trans-lycopene [39], geraniol [40], bixin [26], astaxanthene [31], crocetin [33], gossypol [34], vincristine and vinblastine [41] and perillyl alcohol [42]. Transcripts, corresponding to mevalonate pathway I, were observed to be in 4.94% of the transcripts annotated for terpenoid pathway. Isopentyl diphosphate (IPP) and its isomer dimethylallyl diphosphate (DMAPP), the end-products of mevalonate pathway, are the universal precursors of the terpenoid category [25]. Transcripts related to artemisinin biosynthetic pathway were also observed in pathway annotations; artemisinin, the end-product of the pathway is a proven anti-malarial agent [43]. The annotations of transcripts relating to biosynthetic pathways of linalool, farnesene, bergamotene, capsidiol, gossypol, saponins, oleoresin, isopimaric acid, phytoalexins and sesquiterpenoid phytoalexins suggest that they might provide the plant with either anti-microbial or insect/herbivore defense. The other transcript annotations related to biosynthetic pathways include those of phaesic acid, palunotol, gibberelins and fenchol.

Annotations from other secondary metabolite pathways also provide us information about certain phytochemicals (Additional file 3). 4-coumarate-CoA ligase transcripts, which were predominantly observed, are intermediates in a lot of metabolic pathways, indicating their pivotal roles in plant metabolism. A major chunk of the flavonoid biosynthetic pathway transcripts (36.56%) was contributed by transcripts annotated as 4-coumarate-CoA ligase. Transcript annotations from scopoletin biosynthesis (16.49%) were also found to occur. Scopoletin is known to be involved in plant defense mechanisms [44]. Myricetin, an intermediary metabolite from the observed syringetin biosynthetic pathway, is known to possess anti-oxidative and anti-diabetic properties [45]. Transcript annotations related to anthocyanin metabolism (known for coloration) include rose anthocyanin, shisonin, pelargonidin, and gentiodelphin. Leucopelargonidin and leucocyanidin biosynthetic pathway, precursor to leucodelphinidin biosynthesis, was also noticed in the annotations. We also observed transcripts corresponding to chalcone 2'-*O*-glucosyltransferase and aurone which are known for providing yellow coloration. Antitumor properties might also have been obtained from the observed coumarin [46] and quercetin [47] biosynthetic pathways. Insect resistance could have also been rendered by the presence of glycosyl transferases, pinobanksin and glyceollin biosynthetic pathways. Other general pathways to which the transcripts showed similarity include flavonol biosynthesis I, isoflavonoid biosynthesis I and II.

### Gene ontology (GO) annotation

**Figure 4:**
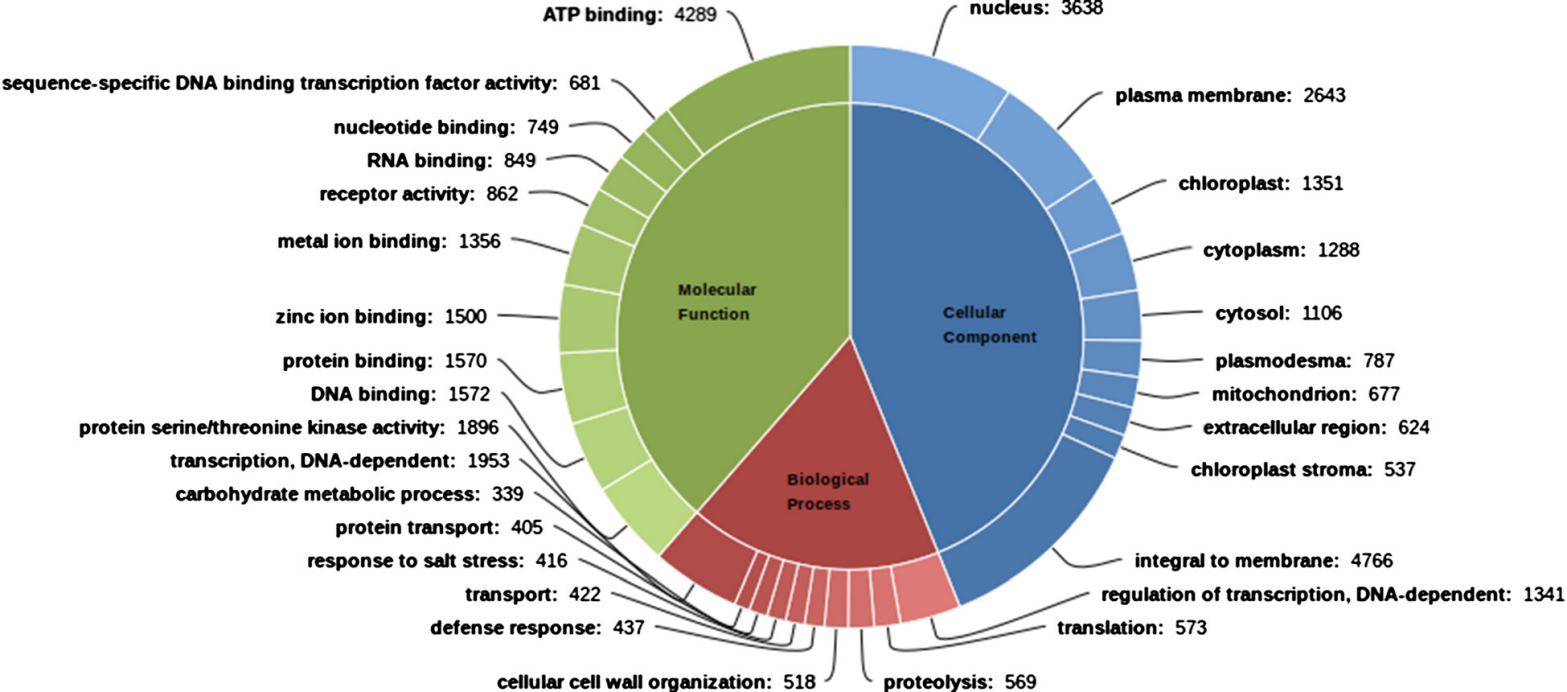
**GO Classification.** GO terms were derived based on the similarity search with Swiss-Prot database. The top 10 GO terms in Cellular Component, Molecular Function and Biological Process are displayed.

The Swiss-Prot database annotation covered 38.25% of the transcripts and GO terms were derived based on the annotation information (Additional file 4). The three categories of GO Cellular component, Molecular function and Biological Process were represented by 27,871, 38,886 and 31,671 terms respectively (Figure 4).

In the Biological Process category, classes related to *DNA-dependent transcription* (6.1%) and *DNA-dependent regulation of transcription* (4.2%) were observed to be occurring most frequently. *Defense response* was represented in many a number of pathways from pathway annotations. *C. pictus* is commonly known for its insect resistance properties and is a common factor in herbal plants, which was reflected in the occurrence of *defense response* among the top Biological Process category. In the Molecular Function category, *ATP binding* (11.02%) was found to be the most abundant class. The most frequently occurring GO terms within Cellular Components include *integral to membrane* (17.1%), *nucleus* (13.05%) and *plasma membrane* (9.4%).

### KOG annotation

**Figure 5:**
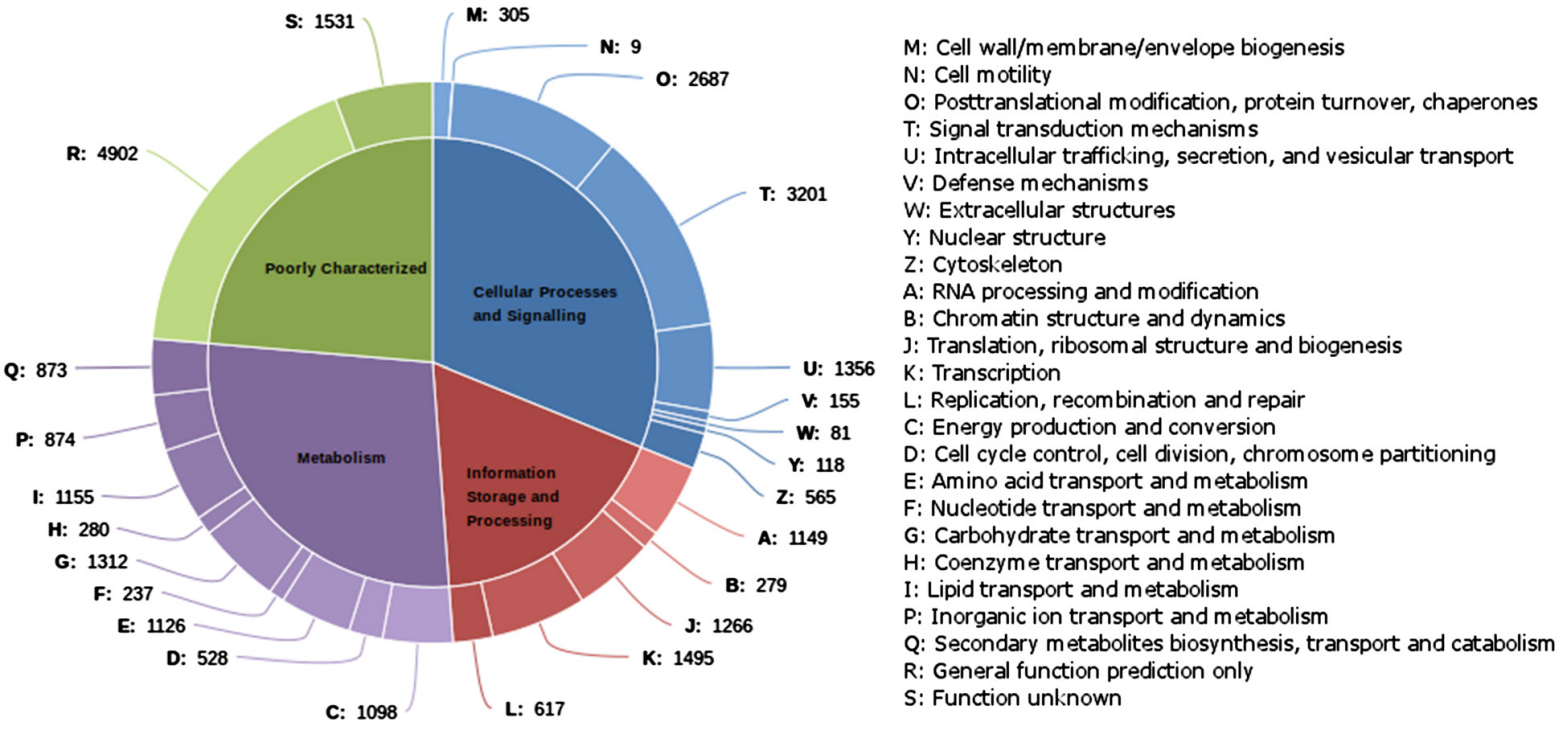
**KOG Functional Classification.** 44.4% (24,424) of the transcripts were annotated against the KOG proteins and were assigned KOG functional categories.

The eukaryotic clusters (KOGs) present in the Cluster of Orthologous Groups (COG) database are made up of protein sequences from *Arabidopsis thaliana*, *Caenorhabditis elegans*, *Drosophila melanogaster*, *Homo sapiens*, *Saccharomyces cerevisiae*, S*chizosaccharomyces pombe* and *Encephalitozoon cuniculi*. The KOG proteins from the eukaryotic clusters were used to annotate 24,424 transcripts and with the help of the annotations, we were able to assign KOG terms to each annotation (Additional file 5). The KOG classifications with multiple assignments were individually assessed and assigned to transcripts (Figure 5).

*Cellular Processes and Signalling* (31.16%) was found to be the major category from the KOG classifications, of which *Signal transduction mechanisms* were found to be prominent (11.07% of the total KOG classifications) followed by *Post translational modification, protein turnover, chaperones* (9.87%) and *Intracellular trafficking, secretion and vesicular transport* (4.98%). In the Information Storage and Processing category, *Transcription* (5.49%), *Translation, ribosomal structure and biogenesis* (4.65%) and *RNA processing and modification* (4.22%) were observed to be highly occurring. In the metabolism category, the frequently observed classes were *Carbohydrate transport and metabolism* (4.82%), *Lipid transport and metabolism* (4.24%), *Amino acid transport and metabolism* (4.13%), *Energy production and conversion* (3.7%). Our focus on the secondary metabolite transcripts and a fair representation of *Secondary metabolites biosynthesis, transport and catabolism* transcripts in KOG classification (3.2%) further attests the data integrity both at sequencing as well as analysis levels. From the poor characterized annotations, *General function prediction only* represented 18.02% and *Function unknown* represented 5.62%, which is quite expected since *C. pictus* is remotely similar to the organisms originally present in the eukaryotic KOG database.

### Pfam annotation

**Figure 6:**
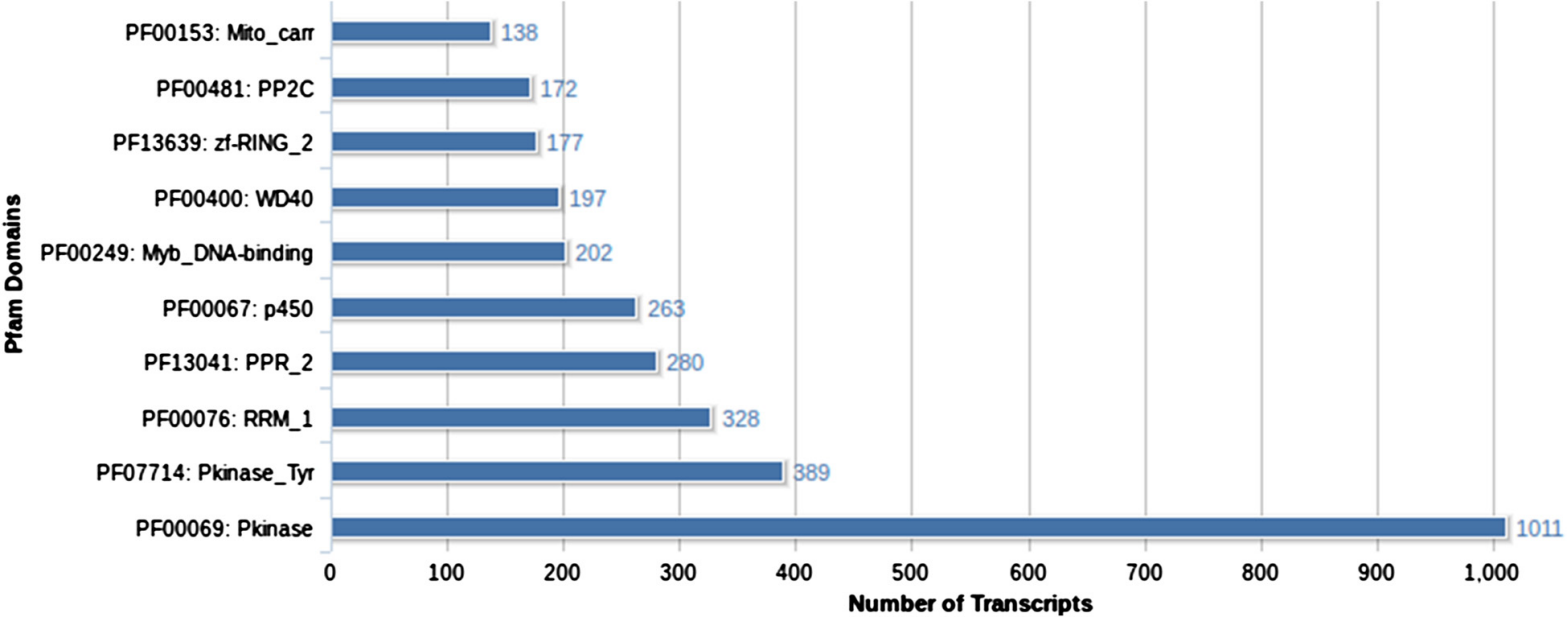
**Top 10 Pfam domains represented in InterProScan transcript annotations.** Pfam Domain annotations were obtained from InterProScan and the top 10 domain annotations were represented in the chart.

Using InterProScan, 25,973 transcripts were annotated against Pfam domains (Additional file 6) and the highly occurring Pfam domains were plotted as a bar chart (Figure 6). The aim of this approach was to identify similarity at domain level, where the proteins have little similarity at sequence level but might share conserved structural domains.

Protein Kinase (Pkinase) domain along with Protein Tyrosine Kinase (Pkinase_Tyr) domain were represented the most in transcripts indicating strong signal transduction mechanisms. WD40 repeat domains which also have significance in signal transduction mechanisms were also observed. Myb domain (Myb_DNA-binding) annotations, significant for being transcription factors with a wide range of functions, were observed in Pfam transcript annotations and corresponded to the observance of a lot of Myb class proteins from Swiss-Prot annotations: MY1R1, MYB06, MYB08, MYB1, MYB2, MYB32, MYB38, MYB4, MYB44, MYB5, MYB86, MYBA1, MYBC, MYBF and MYBP. The other frequently occurring domain was Cytochrome P450 (p450) which mediates oxidation of organic substances. RNA recognition motif (RRM_1), Pentatricopeptide repeats (PPR_2), Mn++ or Mg++ dependent protein serine/threonine phosphatase domains (PP2C), Mitochondrial carrier domains (Mito_carr) and Zinc-finger related RING protein domains (zf-RING_2) were also highly represented in transcript annotations.

### Final annotation table

Even though individual database annotations were used to interpret findings, a final annotation table was obtained in order to arrive at a single best annotation for each transcript. After deriving the best annotation for each transcript from multiple databases (Additional file 7), the final annotations comprised 17,482 (31.78%) transcripts from Swiss-Prot database, 1,041 (1.89%) transcripts from PlantCyc database, 11,768 (21.39%) transcripts from KOG proteins database, 7,243 (13.16%) transcripts from TrEMBL database, 317 (0.58%) transcripts from GenBank Viridiplantae nucleotide sequences and 188 (0.34%) transcripts from Pfam database (Table 3). TrEMBL initially had the highest share of annotations. However, in the final annotation table, major shares of the results were distributed among the well annotated databases (Swiss-Prot and KOG).

**Table 3:** Annotation Statistics

**Database**	**Number of transcripts annotated**	**Percentage of transcripts annotated**
**Swiss-Prot**	17,482	31.78%
**PlantCyc**	1,041	1.89%
**KOG**	11,768	21.39%
**All GenBank (Viridiplantae) mRNA sequences**	317	0.58%
**TrEMBL**	7,243	12.17%
**Pfam**	188	0.34%
**Total**	38,039	69.15%

We observe that some of the transcript annotations were represented as predicted or hypothetical. The following terms were found in the annotation: *Probable* (2,071, 3.76%), *Putative* (679, 1.23%), *Unknown* (18, 0.03%), *Hypothetical* (13, 0.02%) and *Predicted* (1,550, 2.81%). However, the number of such instances is very less, considering that it is a non-model plant from Costaceae family.

### Mapping reads, calling variations and quantification of transcripts

Alignment statistics were reported from the SAM format alignment files using custom Perl codes (Table 4).

**Table 4:** Alignment Statistics

**Category**	**Statistics**
**Total Reads**	41,104,418
**Reads Aligned**	37,388,868
**% Reads Aligned**	90.96
**Reference Sequence Length (in bases)**	48,190,986
**Total Reference covered (in bases)**	47,955,274
**% Total Reference covered**	99.51
**Average Read Depth**	54.57

Large number of the reads (91%) aligned back to the transcripts as expected (Table 4). Due to low expression of certain transcripts, the reads belonging to them might be either partially assembled or left out completely during the assembly process. This leads to a small fraction of reads unused during the assembly process. In our case, 9% of the reads did not align back to the transcript reference sequences. Post-processing the SAM file using SAMtools and on further filtering, resulted in 76,893 SNPs (Additional file 8).

**Figure 7:**
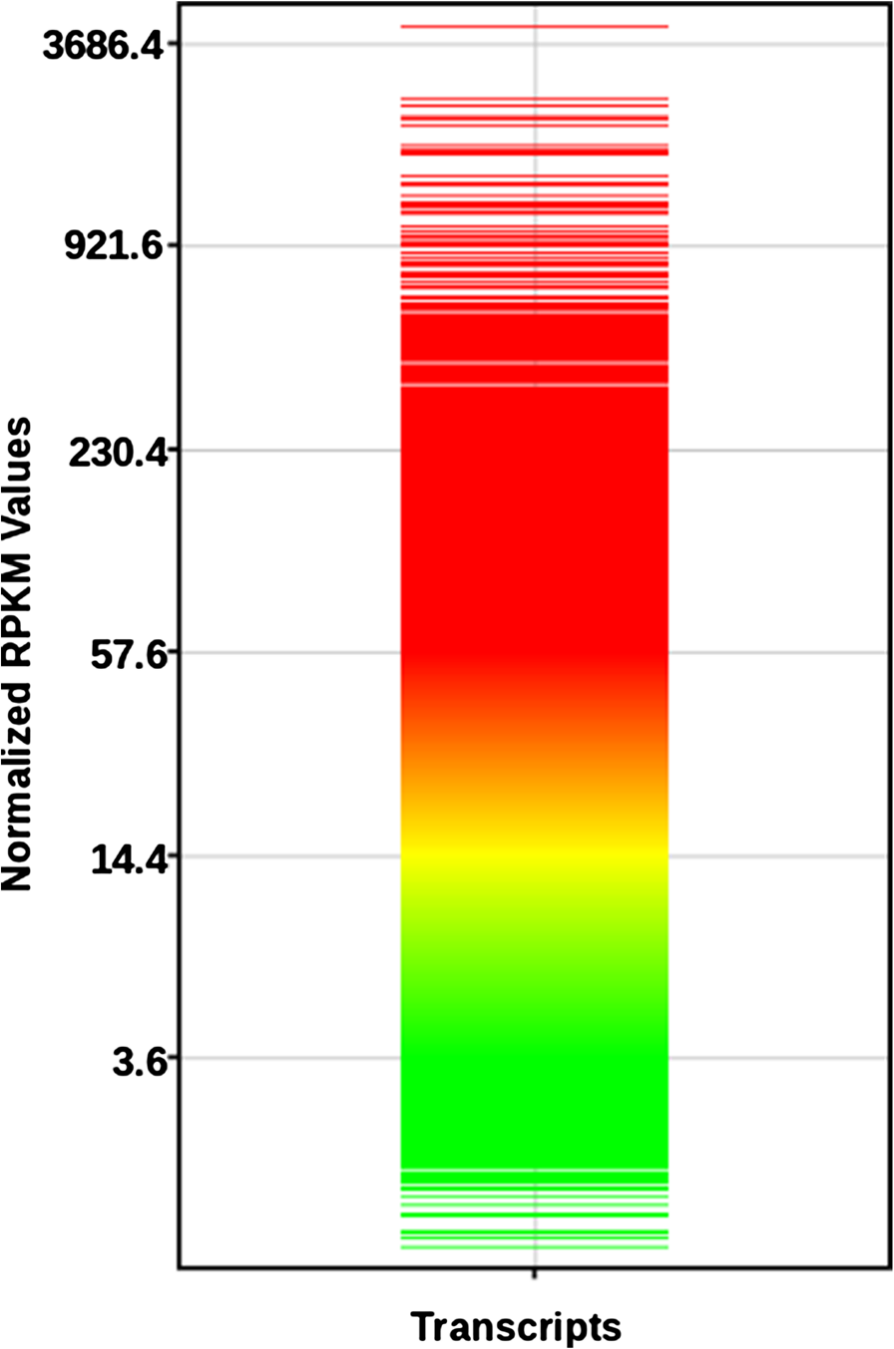
**Expression profile of the transcripts.** The colors ranging from red to green indicate the expression levels from high to low.

An expression profile of the transcripts was created using Agilent's GeneSpring (Figure 7). The transcript with the highest expression levels from the annotation was found to be a *Cell wall hydroxyproline-rich glycoprotein* (Extensin). The other protein annotations which were part of the top 10 highly expressed transcripts include isoforms from Ribulose bisphosphate carboxylase small chain (Chloroplastic), Polyubiquitin 4, isoforms of Chlorophyll a-b binding protein (Chloroplastic), Photosystem I reaction center subunit V (Chloroplastic) and FOG Zinc Finger proteins. There was a putative protein as well among the top 10 highly expressed transcripts. Most of the highly expressed transcripts belong to the class of housekeeping genes. The transcripts which showed lower expressions belonged to either uncharacterized or probable (predicted) class of proteins. However, there was one transcript which showed match to *Auxin response factor 1* from the low expressed transcripts.

### Validation of assembled transcripts

**Figure 8:**
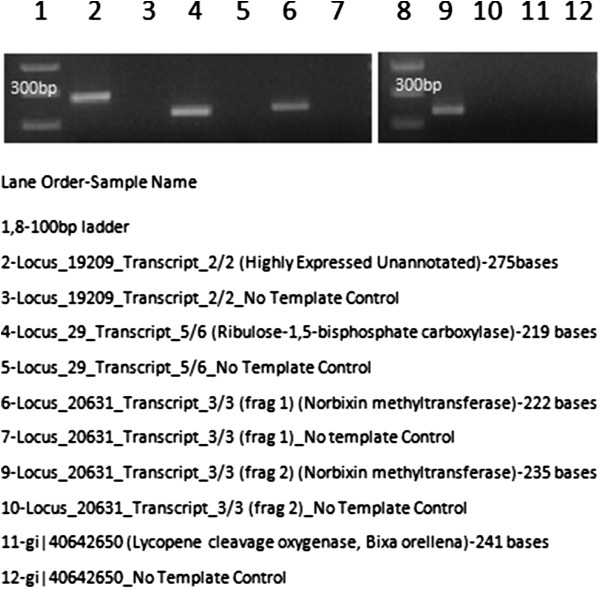
Validation of assembled transcripts.

Validation of the assembled transcripts was performed for two high copy genes viz Ribulose bi phosphate Ribulose-1,5-bisphosphate carboxylase and an unnannotaed transcript and two genes of biological significance viz. Putative norbixin methyltransferase and Lycopene cleavage oxygenase (Bixa orellana). All genes gave amplicons of expected sizes (Figure 8). Lycopene cleavage oxygenease which was not detected by transcript assembly was also not detected by RTPCR using primers from a related species for the same gene (See Supplementary data Additional file 9).

### SSR identification

Short Sequence Repeats (SSRs) are short repeat sequences of 2–6 bases which are important molecular markers in a wide range of genetics and genomics applications. A total of 8,482 SSRs were identified in 7,049 transcripts (Additional file 10). More than one SSR was found to be in 1,126 transcripts. Compound SSRs were observed to be 623 in number. Trinucleotide SSRs were the most abundant accommodating 40.27% of the identified SSRs, followed by tetranucleotides (14.89%) and dinucleotides (10.9%) (Table 5).

**Table 5:** Identification of SSRs using MISA

**Unit size**	**Number of SSRs**
2	1273
3	4663
4	1725
5	381
6	440

### Similarity-search among other anti-diabetic plant resources

After filtering the BLAST results using cut-offs mentioned in the methods, 13 out of 18 sequences from *C. pictus* were represented in the assembled transcripts. Four tRNA partial sequences and a RPB2 partial gene sequence did not match with the transcripts. The results also showed that *C. pictus* is more similar to *Costus speciosus*, another plant with anti-diabetic properties from the same genus (Additional file 11).

### HPLC analysis

**Figure 9:**
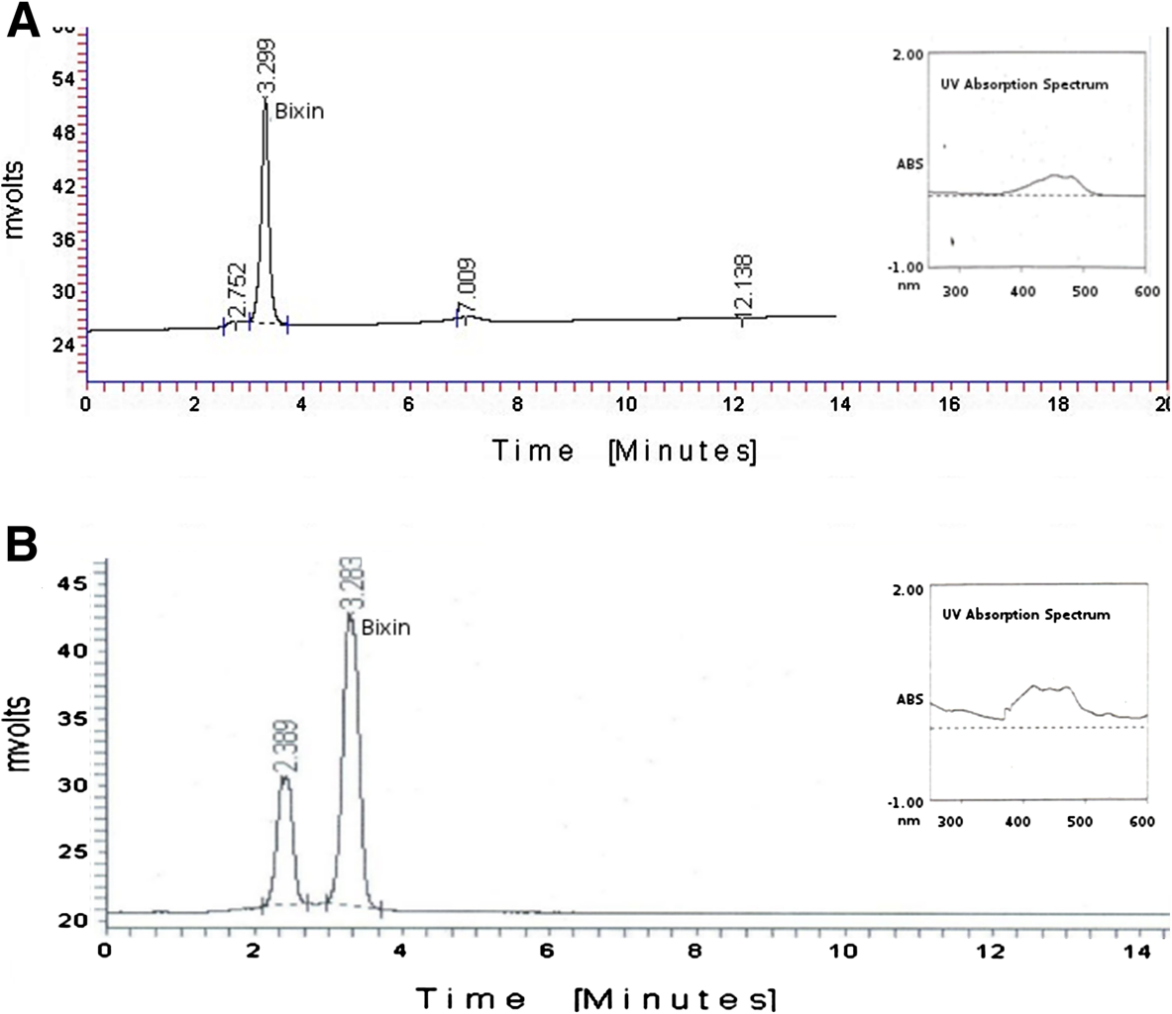
**Chromatograms from HPLC. A**) Chromatogram of standard bixin along with UV-visible absorption spectrum in the eluting solvent (inset). **B**) Chromatogram of *C. pictus* methanolic extract along with UV-visible absorption spectrum in the eluting solvent (inset).

High Performance Liquid Chromatography (HPLC) was used to confirm the presence of Bixin in *C. pictus* methanolic extract. UV-visible absorption spectrum of both standard bixin and the leaf extract was recorded at 444 nm. The chromatograms of the standard bixin and *C. pictus* methanolic extract recorded peaks corresponding to bixin (Figure 9).

## Discussion

Transcriptome wide studies on a variety of organisms have recently been conducted on a large scale, following the revolution introduced by the emergence of Next Generation Sequencers. Whole transcriptome sequencing using an Illumina GAIIx sequencer and analysis of the *C. pictus* plant leaves were reported for the first time in this study, in order to understand molecular signatures related to the anti-diabetic principles. We obtained about 3.2 Gb of raw sequence data, which was processed and *de novo* assembled into contigs and further into transcripts. *De novo* assemblies are highly dependent on k-mer lengths. In general, plant assemblies are very hard and difficult owing to the complex gene contents, higher ploidy, higher rates of repeats and heterozygosity [48]. Longer k-mers are advantageous in distinguishing repeats from real overlaps [49] and are accurate, and in general suit the assembly of highly expressed transcripts [50] while shorter k-mers are preferred for assembly of low expression genes. To balance between higher accuracy from longer k-mers and better assemblies for low expressed genes from short k-mers, we ran multiple assemblies to arrive at an optimal k-mer length for a better assembly. Specific care was taken to remove adapters and low quality sequences from reads, such that a high quality assembly is obtained (Table 1). The N50 value of the assembled data was comparable to other plant transcriptome assemblies indicating a high quality assembly (Table 2).

The complete and accurate transcriptome assembly of plants is difficult and is limited to the currently available *de novo* assembly tools. Hence, in our study, a single transcript might be present redundantly as multiple isoforms or in multiple fractions and some of the transcripts might have been lost during the assembly due to low coverage. For instance, 4-coumarate-CoA ligase is present redundantly in multiple copies, whereas transcripts encoding lycopene cleavage dioxygenase - an important component of the bixin biosynthetic pathway were not observed at all. Nonetheless, once newer efficient assembly tools with improved algorithms are developed in the future, the publicly available raw data can be re-used to create a better transcriptome assembly. The attempt was made to not only computationally characterize the transcriptome, but also to derive molecular clues to the medicinal properties of the plant. We were successful in establishing a relationship of the anti-diabetic property with the genetic makeup. Interpreting high-throughput data is a challenging aspect and we have suggested ways to analyse and interpret a plant transcriptome. It has been estimated that 15 to 25% of the plant genome specifies pathways of natural product biosynthesis [51]. The high number of transcripts that have been annotated as secondary metabolite profiles from *C. pictus* is a clear indication of the genetic complexity of the species.

Our primary focus has been to understand the transcripts involved in biosynthesis of the anti-diabetic principles. The surprising presence of high number of transcripts corresponding to bixin, norbixin and geraniol indicate possible involvement of these active constituents in the plant's anti-diabetic activities (Figure 3). The presence of the transcript for Putative norbixin methyltransferase further confirms these findings (Figure 8). *Bixia orellana* (Annato) is currently reported to be the sole source of the natural pigment bixin [52], but our findings on the presence of significant levels of bixin in *C. pictus* leaves suggests that the leaves could be used as an alternative source of Bixin for commercial supply. Bixin and norbixin from Annato has been indicated to activate Peroxisome Proliferator-Activated Receptor α (PPARα), which in turn stimulates adipocyte differentiation and increases the insulin dependent glucose uptake in differentiated 3T3-L1 adipocytes [26]. The identification of bixin synthase transcripts from our current annotations was corroborated from results suggesting presence of bixin from HPLC (Figure 9). Geraniol activates both PPARγ and PPARα thereby improving hyperlipidemia and glucose uptake [27]. ABA is another notable terpenoid observed in our transcript annotations which has anti-diabetic, anti-inflammatory, anti-obesity and immuno-modulatory properties. ABA was observed to be an endogenous stimulator of insulin release from human pancreatic islets [28]. ABA is also known to significantly increase the expression of PPAR and its associated genes CD36 and aP2 [29]. An earlier report states that the administration of aqueous extract of *C. pictus* leaves in rats have significantly reduced the levels of triglycerides and cholesterol, along with reduction in glucose [7]. Purified methyl tetracosanoate from *C. pictus* treatments in cells at 18 hours exhibited PPARα expression equivalent to rosiglitazone (50 lM) and the methanolic extracts exhibited anti-diabetic activity as well as anti-adipogenic activity [8]. It is possible that the reduction in the levels of glucose, triglycerides and cholesterol might have occurred through the activation of both PPARγ and PPARα pathways by ABA, bixin, norbixin or geraniol. These terpenoids might act as insulin sensitizers in a way similar to thiazolidinedione drugs. Ginger (*Zingiber officinalis*), a taxonomically closely related species, is shown to be effective against the development of cataract, a diabetic complication, in rats through its anti-glycating potential [53]. *C. pictus* is also reported to be an anti-glycation agent [9], which might be due of the presence of geraniol and farnesene derivatives (geranylgeranyl, farnesylacetone, geranylgeranyl octadecanoato, geranylgeranyl formiate and geranylgeranyl acetate) which were observed to inhibit glycation and Advanced Glycation End-product (AGE) formation [52], thereby inhibiting certain diabetic complications. Aldose reductase, an enzyme of polyol pathway, is involved in diabetic complications and docking studies show that citral (a mixture of geraniol, geranial and neral) as well as geraniol inhibit aldose reductase activity [54]. The frontline anti-diabetic drug “Metformin” also known as “Dimethylbiguanide” was developed from a plant based molecule from *Galega officinalis*. Current leads reported for the first time from *C. pictus* might also emerge as a powerful anti-diabetic and anti-glycation agents, if researched further. Validation at the biochemical, cellular and pharmacological levels will supplement the transcriptomic observations.

Reactive Oxygen Species (ROS) are beneficial to the organism and they are involved in signalling pathways and are also toxic to pathogens [55]. But an increase in ROS may be observed in many metabolic disorders and are harmful. Oxidative stress and an increase in ROS are common events accompanied with type II DM. In fact, ROS have been shown to have a casual role in insulin resistance and a decrease in ROS suppressed the insulin resistance activity [56]. Hence, it is common to note that most anti-diabetic herbal remedies are also potential anti-oxidants. The anti-oxidant properties of *C. pictus* have already been reported [9]. ROS may have potential role in either cell proliferation or cell death which is dependent on the intensity/location of oxidative burst and also the anti-oxidant activities. In cancer cells, an increased constitutive oxidative stress supports tumor growth and protects the tumor from pro-apoptotic signals promoting tumor progression [55]. A reduction in oxidative stress leads to suppressing tumors. *C. pictus* is also shown to have anti-oxidant as well as antitumor properties [10]. A number of secondary metabolites were reported in this study which corresponded to anti-oxidant and antitumor properties of *C. pictus* leaves. Compounds classified as anti-oxidants generally reduce the oxidative stress, but under certain conditions they act as pro-oxidants. For instance, under non-physiological conditions, although norbixin, a precursor of Bixin was able to protect DNA from damage by ROS, it might also create circumstances that amplify damaging oxidative signal, unless some other anti-oxidant comes to the defence [57]. This leads us to suggest that a single isolated compound might not have the desired effect and might also turn out to be toxic while promoting DNA damage as a pro-oxidant. Hence, a combination of plant compounds at optimal dosage is probably necessary for a beneficial effect on a system.

*C. pictus* plants are known for their excellent insect resistance potentials. They are also reported to have anti-microbial properties [9]. The same is supported by the secondary metabolite pathway annotations. It should be noted that secondary metabolites from plants are generally expressed in minimal quantities by the plants, in contrast to the expression of primary metabolites. The fragmentation of the mRNAs during library preparation could lead to the potential loss of whole or part of some important genes, if their expression is very low. Low expression also means that considerable sequence coverage will not be available and the fragmented sequences might not be assembled into complete transcripts. Hence, we chose to include any pathway hit in the annotation, even if only fewer enzymes were captured in sequencing. For instance, lycopene cleavage dioxygenase which converts lycopene to bixin aldehyde was cloned in *Escherichia coli* and it subsequently activated bixin biosynthetic pathway [51]. In our study, we did not observe transcripts corresponding to lycopene cleavage dioxygenase enzyme, whereas transcripts corresponding to the other two enzymes bixin aldehyde dehydrogenase and norbixin carboxyl methyltransferase were observed. One possibility could be that the transcript was not expressed at adequate levels and might have been lost during the *de novo* assembly or during cDNA fragmentation before sequencing. The other possibility might be the presence of an alternate precursor for bixin biosynthesis. At this level, we could only attribute these reasons for the missing transcripts. Critical annotations from GO (Figure 4) and KOG (Figure 5) supported evidences of signal transduction mechanisms, resistance properties, DNA binding functions and defense mechanisms. Pfam annotations (Figure 6) abounded with Protein kinase domains. There is evidence that *C. pictus* initiates an insulin secretory response by increasing Ca^2+^ influx through VGCC in mouse and human islets cell cultures [5]. In human granulocytes, ABA has been shown to bind to plasma membrane through a pertussis toxin (PTX)-sensitive receptor-G protein complex, which leads to an increase in cAMP, activation of protein kinase, phosphorylation of the ADPRC CD38 with cADPR overproduction, eventually leading to an increase of the Ca^2+^[29]. The presence of ABA biosynthesis transcripts (Figure 3) in the present study involving pathway annotations could be functionally correlated with the anti-diabetic activity of *C. pictus* possibly through activation of protein kinases.

The expression study gives us some clues about the assembly. The transcripts with least expression values could either be novel genes of interest with very low copy numbers or they could be mis-assemblies which did not find any similarity with the sequence databases. Apart from just annotating the data, we have also mined the data for other information like SNPs and SSRs which will be invaluable, especially because *C. pictus* is a non-model plant without genome sequences being available. The reported SNPs and SSRs could be used as molecular markers for the construction of genetic linkage maps in the future. Substantial quantities of oxalate content and oxalate oxidase activity were reported in fresh leaf extracts [58]. The annotation results, however, did not pick up oxalate oxidase or oxaloacetate acetylhydrolase (enzyme involved in conversion of oxaloacetate to oxalate) in our transcripts. Our analysis indicates only the presence of malate dehydrogenase, the enzyme involved in the conversion of malate to oxaloacetate.

## Conclusions

We are reporting for the first time, solid molecular signatures (transcripts related to bixin, ABA, and geranial and geraniol biosynthesis) for the anti-diabetic properties of *C. pictus* leaves and are also providing vital clues related to the other phytochemical functions like antitumor, anti-oxidant, immuno-modulatory, anti-microbial and anti-malarial properties through the secondary metabolite pathway annotations. Further, an analytical proof of presence of bixin in *C. pictus* leaves is provided through HPLC. We believe that this data will be of immense help to researchers working in the treatment of DM using herbal therapies. Even though our focus was on transcripts relating to anti-diabetic principles, we have limited clues about the role of several other transcripts with no assigned function as of now. They may modulate an anti-diabetic role in conjunction with the major metabolites or conversely, they may exert adverse reactions at cellular level. Advocating whole leaf consumption to diabetic patients may not be advisable considering the phytochemical complexity, as indicated by the transcriptome profile. Hence, a thorough clinical research of the biochemical and physiological properties of *C. pictus* leaf extracts may be warranted before recommending it for large scale usage by hyperglycemic individuals.

## Methods

### Sample collection and preparation

Fresh *C. pictus* leaves (fifth leaf from the bud) were collected from a domestic garden of one of the authors from Bangalore, India and brought to the laboratory in ice. RNA was extracted from the leaf sample frozen in liquid nitrogen, using Agilent Plant RNA isolation mini kit (Product No; 5188–2780) and was quantified using Nanodrop. QC was performed using Agilent's Bioanalyzer. RNA Integrity Number (RIN) was observed to be 8.2. Transcriptome library for sequencing was constructed as outlined in Illumina's “TruSeq RNA Sample Preparation Guide v2”.

### Sequencing and quality control

Illumina GAIIx was used to generate 73 base paired-end short reads using Sequencing By Synthesis (SBS). Software including Real Time Analysis (RTA), Consensus Assessment of Sequence and Variation (CASAVA) and Off-Line Basecaller (OLB) from Illumina standard pipeline was used to generate short read information in FASTQ format (http://www.illumina.com/support/sequencing/sequencing_software.ilmn). Additional quality control was performed using SeqQC V2.1 (http://genotypic.co.in/SeqQC.html). Accuracy of base calling is reflected in the quality scores and low quality scores usually denote high error probabilities. Low quality bases, if due to errors, will interfere in the assembly process either resulting in mis-assemblies by collapsing repeat regions or fragmentation of contigs by obscuring true overlaps [49]. Hence, quality filtering is very essential in order to arrive at a high quality assembly. The adapters, B tails (CASAVA1.7 User Guide), and other low quality bases were filtered or trimmed using in-house Perl scripts. Thus filtered, high quality reads were used for further analysis.

### *De novo* assembly

*De novo* assembly of reads into contigs was performed using De-brujin graph based assembler Velvet 1.1.07 – http://www.ebi.ac.uk/~zerbino/velvet/[49]. Parameters like observed insert length and expected coverage were estimated using an initial draft assembly. The final assembly was generated with the parameters: k-mer as 47, insert length as 154 +/− 51.6, expected coverage as 5 and coverage cut-off as 'auto'. The contig assembly was followed by a transcriptome assembly with default parameters using Oases 0.2.01 - http://www.ebi.ac.uk/~zerbino/oases/[50]. Transcripts with at least 200 bases were considered for further analysis. In-house Perl scripts were used to compute assembly statistics to assess the quality of assembly.

### Functional annotation

Annotation of novel transcriptomes is a challenging task, hence, various databases were chosen to extract the maximum possible information based on sequence and functional similarity. The information collected include Plant Pathway information (PlantCyc Enzymes database v2.0 (http://www.plantcyc.org)), protein level sequence similarity information (UniProt: Swiss-Prot and TrEMBL databases downloaded as of 21st March 2012 [59]), nucleotide level sequence information (Viridiplantae mRNA database from GenBank downloaded as of 14^th^ March 2012), Clusters of Orthologous Groups (COG) functional classifications (KOG proteins from COG database downloaded as of 9^th^ April 2012 [60]), and information on protein domains for distantly related proteins which do not have similarity at sequence level (Pfam database v26.0 [61]).

Similarity search was performed using locally installed BLAST+ v2.2.25 software [62]. The transcripts were subjected to similarity search against protein and nucleotide sequence databases using blastx and megablast respectively at an e-value cut-off of e-5. BLAST annotations were filtered using either subject or query coverage (>30%) and sequence identity (>50% for megablast and identity >30% for blastx). Terpenoids along with other secondary metabolites are known to be involved in a number of therapeutic remedies; hence these metabolites were critically examined from the annotations. InterProScan v4.8 - http://www.ebi.ac.uk/Tools/pfa/iprscan/[63] was used to identify possible protein domains in the transcripts.

### Validation of transcripts

Primers were designed spanning ~200 bases or more of the assembled transcripts (See supplementary data). 1 ug of total RNA from *C. pictus* was converted to cDNA using Affinityscript Reverse Transcriptase from Agilent Technologies by using Oligo dT primers. cDNA was dissolved in 50 ul nuclease-free water and 2 ul was used as template for each qRT-PCR reaction. qRT-PCR for each primer pair was carried out in duplicates on an Agilent technologies Stratagene Max3005p Real time PCR machine using the following conditions.

95C for 10 mins, ( 95C for 30sec, 55C for 1min, 72C for 1min) for 40 cycles followed by 72C for 2mins for final extension. Dissociation curves were generated using 95C for 1min 55C for 30 sec and 95C for 30sec.

### Final annotation table

To obtain a final annotation table, the annotations from each database were analysed using the BLAST scoring system [62] to obtain the best annotation for each transcript. The order of preference for obtaining the best annotation was Swiss-Prot > PlantCyc > KOG. In case, annotation information is unavailable from these three databases, then information from TrEMBL or GenBank Viridiplantae Nucleotide database annotations was used. Pfam domain annotation was assigned, if the transcript was not similar to either protein or nucleotide databases.

### Mapping reads, calling variations and quantification of transcripts

Due to lack of availability of a reference sequence, the assembled transcripts were assumed to be the reference sequence to compute transcript expression levels [20,22,23]. The expression values were used to create an expression profile with the help of Agilent's GeneSpring. The read sequences were aligned against these transcript reference sequences using Bowtie2 v2.0.0-beta5 - http://bowtie-bio.sourceforge.net/bowtie2/index.shtml[64] in end-to-end alignment mode. The alignments were processed for further analysis like variant calling using SAMtools v0.1.7a - http://samtools.sourceforge.net/[65]. A combination of reads showing variation and read depth, along with mapping quality and SNP quality were considered for filtering the SNPs (Additional file 12). In-house Perl scripts were used to compute the alignment statistics. The expression levels of the transcripts were estimated using Reads Per Kb per Million reads (RPKM) normalized measure [66].

### SSR identification

MISA (MIcroSAtellite identification tool - http://pgrc.ipk-gatersleben.de/misa/) was used to identify SSRs. Dinucleotide and Trinucleotide repeats were given a minimum threshold of 6 and 4 repeats respectively. Tetra, Penta and Hexanucleotide repeats were given a minimum threshold of 3 repeats. The maximum distance between two SSRs was specified as 100 bases.

### Similarity-search among other anti-diabetic plant resources

The transcripts were compared with known anti-diabetic plant sequence resources which are found to have little sequence information. Nucleotide sequences of *Costus speciosus* (29)*, Syzygium cumini* (15), *Zingiber officinale (199), Vaccinium myrtillus* (34), *Panax quinquefolius* (237), *Rosmarinus officinalis* (59), *Momordica charantia* (194), *Gynostemma pentaphyllum* (95), *Trigonella foenum-graecum* (47) and also *C. pictus* (18) were downloaded from NCBI GenBank database. Pairwise alignments of *C. pictus* transcripts using megablast against these plant species were performed to observe similarity.

### HPLC measurements

HPLC analysis of the methanolic leaf extracts of *C. pictus* was performed with L-4000 UV detector, L-6200 Intelligent pump and Varian Pursuit C18 5μ column from Hitachi with DataAce workstation to detect the presence of bixin. The working standard concentration was 1mg of bixin (96.5% purity by HPLC; Source: Chromadex, Inc) in 1ml of 1:1 dichloromethane: methanol. The dried methanol extract of *C. pictus* leaves was dissolved in the concentration of 1mg in 1ml of 1:1 dichloromethane:methanol. The solvent system containing 0.1% Trifluoroacetic acid in HPLC water as A and acetonitrile as gradient elution of 50-90% of B over 10 minutes and held at 90% B for 4 minutes was used as the mobile phase and the flow rate was maintained at 5.0 ml/min at a wavelength of 444 nm. The sample was filtered through sodium sulphate and C18 cartridges, after which 10μl sample was injected and calibration curve for bixin was generated.

## Competing interests

The Authors declare no competing interests either financial or non-financial.

## Authors’ contributions

RSA proposed, initiated and led the project, collected literature, interpreted scientific information and assisted in manuscript preparation. VJ participated in sequence assembly, alignments and annotation of the data, submitted data to online databases, drafted the manuscript and also interpreted scientific information. RCM involved in scientific advising and supported technically. MAK assisted in bioinformatics analysis. SA extracted RNA from the initial plant material. SG prepared sequencing library. SPS sequenced the library. SJF assisted in RNA extraction, library preparation and sequencing. NM monitored the entire wet lab work. SM performed HPLC experiment. SNR coordinated sequencing and involved in scientific advising. All authors have read and approved the final manuscript.

## Supplementary Material

Additional file 1 **Venn diagram depicting sharing of transcripts annotated by six different databases.** The Venn diagram shows transcripts unique to each database and which are shared amongst different databases. Click here for file

Additional file 2 **PlantCyc Enzyme Annotations.** The tab delimited table lists the pathway annotations from PlantCyc enzymes annotation. Click here for file

Additional file 3 **Other Secondary Metabolite Annotations.** The document shows the percentage distribution of other secondary metabolite pathway related transcripts observed from PlantCyc enzymes annotation. Click here for file

Additional file 4 **Swiss-Prot Annotations.** The tab delimited table lists the Swiss-Prot annotations leading to Gene Ontology term classifications. Click here for file

Additional file 5 **KOG Annotations.** The tab delimited table lists the annotations from Cluster of Orthologous Groups leading to KOG classifications. Click here for file

Additional file 6 **Pfam Annotations.** The tab delimited table lists the annotations from Pfam protein domains. Click here for file

Additional file 7 **Final Annotation table.** The final tab delimited table lists the best annotation assigned to transcripts after picking the best annotation from individual databases. Click here for file

Additional file 8 **SNPs.** The tab delimited table lists the SNPs obtained after aligning the reads back to the transcripts. Click here for file

Additional file 9 Supplementary data for Validation of assembled transcripts of *C. pictus.* Click here for file

Additional file 10 **SSRs.** The tab delimited table lists the SSRs identified using MISA. Click here for file

Additional file 11 **Similarity search among other anti-diabetic plant resources.** The file provides results of similarity search of the transcripts against GenBank nucleotide sequences from other anti-diabetic plants. Click here for file

Additional file 12 **SNP filtering criteria.** The file provides criteria used for filtering SNPs. Click here for file
