# Supplementary material for: Next generation sequencing and de novo transcriptome analysis of Costus pictus D. Don, a non-model plant with potent anti-diabetic properties
Source: BMC Genomics. 2012 Nov 23;13:663. doi: 10.1186/1471-2164-13-663 (PMC3533581; doi:10.1186/1471-2164-13-663)
Supplement: Additional file 1 — Venn diagram depicting sharing of transcripts annotated by six different databases. The Venn diagram shows transcripts unique to each database and which are shared amongst different databases. [file 1471-2164-13-663-S1.doc]

**Six-way Venn Diagram:**

A six-way venn diagram was constructed to show the sharing of transcripts
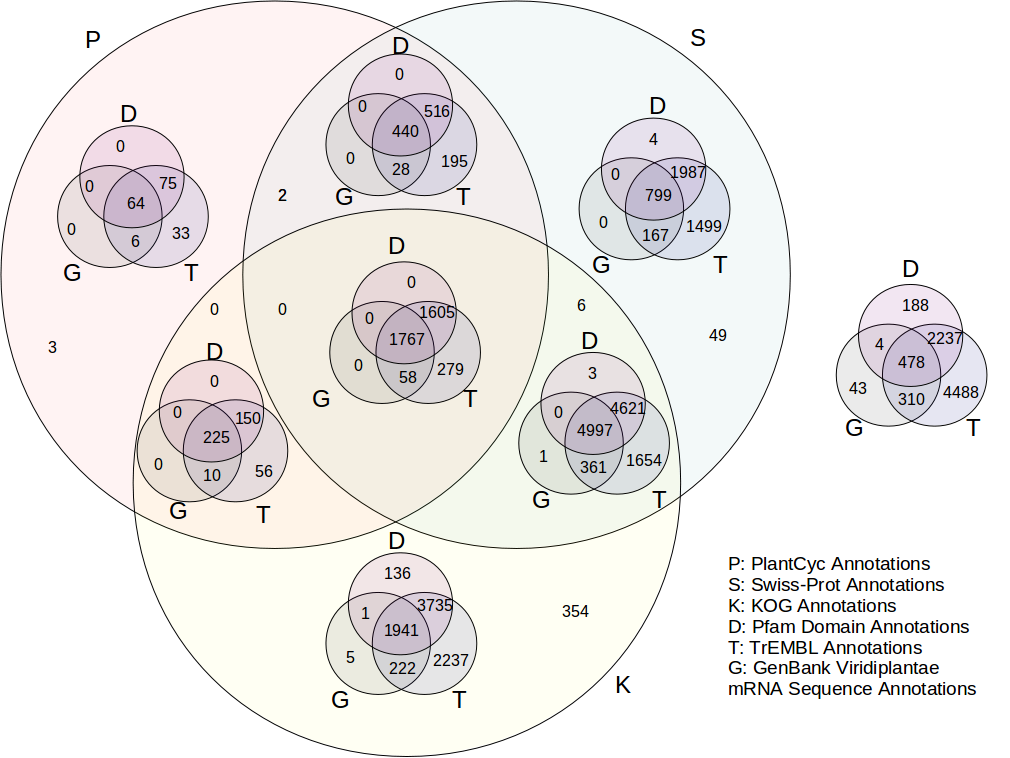
annotated by the six databases.

**Figure: Venn Diagram depicting sharing of transcripts annotated by 6 different databases.** The venn diagram shows transcripts unique to each database and which are shared amongst different databases.

The Venn diagram clearly depicts the number of transcripts which are matching uniquely to a particular database and also the number of transcripts, which are inter-shared among the databases.
