# Supplementary material for: Next generation sequencing and de novo transcriptome analysis of Costus pictus D. Don, a non-model plant with potent anti-diabetic properties
Source: BMC Genomics. 2012 Nov 23;13:663. doi: 10.1186/1471-2164-13-663 (PMC3533581; doi:10.1186/1471-2164-13-663)
Supplement: Additional file 3 — Other Secondary Metabolite Annotations. The document shows the percentage distribution of other secondary metabolite pathway related transcripts observed from PlantCyc enzymes annotation. [file 1471-2164-13-663-S3.doc]

**Other Secondary Metabolite Annotations**

The annotation information from secondary metabolites other than terpenoids are represented as pie-chart in the following figure.


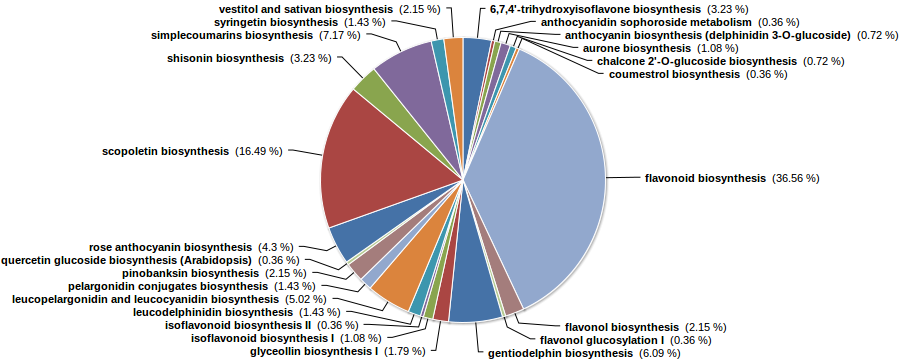


**Figure: Other secondary metabolite annotations.**
