# Supplementary material for: Next generation sequencing and de novo transcriptome analysis of Costus pictus D. Don, a non-model plant with potent anti-diabetic properties
Source: BMC Genomics. 2012 Nov 23;13:663. doi: 10.1186/1471-2164-13-663 (PMC3533581; doi:10.1186/1471-2164-13-663)
Supplement: Additional file 9 — Supplementary data for Validation of assembled transcripts of C. pictus. [file 1471-2164-13-663-S9.docx]

SUPPLEMENTARY INFORMATION:-**Validation of Assembled transcripts of Costus pictus**:

Given that the transcripts were assembled from 73 bases long paired end reads, primers were designed spanning ~200 bases or more, thus allowing us to confirm the assembly based on the size of the product obtained post qRT-PCR. 1 ug of total RNA from C. pictus was converted to cDNA using Affinityscript Reverse Transcriptase from Agilent Technologies by using Oligo dT primers. cDNA was dissolved in 50 ul nuclease-free water and 2 ul was used as template for each qRT-PCR reaction. qRT-PCR for each primer pair was carried out in duplicates on an Agilent technologies Stratagene Max3005p Real time PCR machine using the following conditions: 95C for 10 mins, ( 95C for 30sec, 55C for 1min, 72C for 1min) for 40 cycles followed by 72C for 2mins for final extension. Dissociation curves were generated using 95C for 1min 55C for 30 sec and 95C for 30sec.

qRT-PCR was conducted on 4 predicted genes, 5 fragments as described below.

|  | Transcript id | Annotation | EXPECTED AMPLICON SIZE | OBSERVED AMPLICON SIZE | Ct (Avg of two replicates) |
| --- | --- | --- | --- | --- | --- |
| 1 | Locus_19209_Transcript_2/2 | Highly expressed, unannotated | 275 | ~ 275 | 18.045 |
| 2 | Locus_29_Transcript_5/6 | Ribulose-1,5-bisphosphate carboxylase | 219 | ~ 220 | 14.105 |
| 3 | Locus_20631_Transcript_3/3 (frag 1) | Putative Norbixin methyltransferase | 222 | ~ 220 | 23.065 |
| 4 | Locus_20631_Transcript_3/3 (frag 2) | Putative Norbixin methyltransferase | 235 | ~ 235 | 23.3 |
| 5 | gi\|40642650 | Lycopene cleavage oxygenase  (Bixa orellana) | 241 (This transcript was not detected in NGS data for C. pictus) | None observed | No Ct |

PRIMERS USED:-

|  | FORWARD PRIMER | REVERSE PRIMER |
| --- | --- | --- |
| Locus_19209_Transcript_2/2 | AGCACATTTCGTCACCTTCC | CGGTGTACACACACCCAAAG |
| Locus_29_Transcript_5/6 | TGGCCTCCTCTATGATGGTC | AGGTAGGACAGCGTCTCGAA |
| Locus_20631_Transcript_3/3 (frag 1) | GCCAATTCAAGGATTCAGGA | TTGTATCTCCGGTGGCTTTC |
| Locus_20631_Transcript_3/3 (frag 2) | CCCTTGTGGATCTGTGAACC | TAACACCATTTCCCCTCCAA |
| gi\|40642650 | GAGGAGAGCCGTTCTTTGTG | CCGATTGTTTATGCCTGGTT |

GEL electrophoresis of the products of RTPCR:-


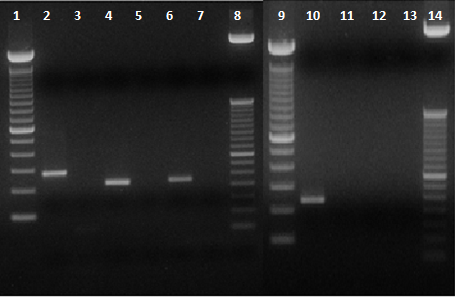


# Lane Order: Sample Name

1. 100bp ladder
2. Locus_19209_Transcript_2/2 (Highly Expressed Unannotated)
3. Locus_19209_Transcript_2/2_No Template Control
4. Locus_29_Transcript_5/6 (Ribulose-1,5-bisphosphate carboxylase)
5. Locus_29_Transcript_5/6_No Template Control
6. Locus_20631_Transcript_3/3 (frag 1) (Norbixin methyltransferase)
7. Locus_20631_Transcript_3/3 (frag 1)_No template Control
8. 50bp Ladder
9. 100bp Ladder
10. Locus_20631_Transcript_3/3 (frag 2) (Norbixin methyltransferase)
11. Locus_20631_Transcript_3/3 (frag 2)_No Template Control
12. gi|40642650 (Lycopene cleavage oxygenase, Bixa orellena)
13. gi|40642650_No Template Control
14. 50bp Ladder

qRTPCR products were resolved on a 2% Agarose gel. Each primer pair is followed by its corresponding No Template Control. Products are loaded in the order 1 to 5, and corresponding product sizes were observed as expected (Table)

ASSEMBLED SEQUENCES OF THE TRANSCRIPTS:-

Forward primer fonts in Red and reverse primer fonts in Green.

>Locus_19209_Transcript_2/2_Confidence_0.067_Length_488 (highly expressed but no annotation)

AATTATAACAAACCCTCCACATCTGGAAATCCTTCACACGAATCCTAAGAACGGAACGAAACAAGCATTATTACGGTTTGTCTCAACTGAGATGCAGGCGTACACCAACCAATACAAGGAGCAATATAAATAAGCACATTTCGTCACCTTCCCCTCCTATGGCTCCGGGTGCACTTCAACGGGAGGGTAACCTGGGTGCCCTGGCTTCGAGTACTTGTGCCAGTGGGGCAATGGCTTCTTGTCGAGTAGCGGGAAGTGGATTTTGGGGTGCGGGTGGTCGTGGTGCTCCAAAAGTGGATGCTGGTAGTGCGGAACCGGAGTCGGTTTGTAGACTGGCGCCGAAGGATGGTGGTAGTATCCTCCTGTCGGTGGACTGTAAGAAGGCGG**CTTTGGGTGTGTGTACACCG**GCGCCGGCGGGTTGTAATATCCCCCTGACGGCGGCGTGTAGGTAGGAGGTGAGTGCACAGGATGGGTGTAGGTAGGTGGTG

>Locus_29_Transcript_5/6_Confidence_0.400_Length_1228 (second highly expressed transcript / ribulose-1,5-bisphosphate carboxylase)

CGAATCGAATCAATCAATCAATCAATTTATGGCTTCTTCCATGATGGTGGTGTACACCGCCGCCACCGCGGCCCGGGCTTCCCCGTCGCAATCCAGCATGGTGGCCCCCTTCACCGGCCTCAAGTCCACCTCCGCCTTCCCCCTCACCAGGAAGCCGGCCAACGCCGACCTCTCCCACCTCCCCAGCAACGGCGGCAGAGTCTGGTGTGGAGGCTAAATTCCACTGATCAAGCAAGCAGGGAAGGAAGAGGGAACGAGAGATGGCCTCCTCTATGATGGTCTCCTCCACCGTGGCCCGGGCTTCCCCGGCGCAATCCAGCATGGTGGCGCCCTTCACCGGGCTGAAGTCCGCCGCCGCCTTTCCCGCCACCAGGAGGGCCAACGCCGACCTCTCCCACCTCCCCAGCAACGGCGGCAGAGTCCAGTGCATGAAGGTGTGGCCGATCGAGGGGAAGAAGAAG**TTCGAGACGCTGTCCTACCT**TCCGACCCTGTCGGAGGAGGCTCTGCTGAAGCAGATCGATTACCCTGCTCCGCTCCAAGTGGGTTCCCTGCTTGGAGTTCAGCCACGAGGGGTTCGCGTGTGGAGGGAGCACACCGCTCGCCGGGGTACTACGACGGGCGGTACTGGACGATGTGGAAGCTGCCCATGTTCGGGTGCAACGACGCGGTGCAGGTGGCGAAGGAGGTGCACGAGTGCAAGAAGGAGTATTCCCAACGCCTTCATCCGCATCATCGGCTTCGACAACGTCCGCCAAGTGCAGTGCATCAGGTTTCATCGCCTTCAACCCCCGGGCCACATAGAATATGGAATATTGAAAGCAATTAATCAAATCACATATAATATATATTATGTATAATAATTAAGTACTGTATCGAATTTGCAAGTTCCGATCGGTTTGGTTCGGCCGGTTCGGTTTCATTCGGGTTTATTGATCGGTTTAATATGCGAATTATATTCTGGCTTACGGAAACAGGCGAGAATGTACTGCGCTGCAGCTGCTGCGGATGTTTTTGTAAGGGGGCATAATATGTTTGCTTACTACTACTGTTGTATGCTTTGCAAGTTATTCATGAACGAACGAACCGCTTATCCTTCTCCTTGCTGTTTACTCTTTCTTGCTCAACTCACTGACCCAGGCCCAGTCCCAGGCCCATGATCCATGGACAATGGTCCAATCGCCTCAGACTGAGATCTGCGAAGAGTAAGTTTATTGGAAGGGGTCAGCGG

>Locus_20631_Transcript_3/3_Confidence_0.600_Length_1334 (norbixin methyltransferase)

CAATCTCGTCCCCTCAAGTGTGCTTAGCTTAATTACGAGCATGAAGGTAGACCAAGTCCTTCACATGAACGGTGGTACTGGGGACACCAGCTACTCTGCCAATTCAAGGATTCAGGAGAAAGTAATCCTGATATTGAGGCCAATACTGAAGAATTCCATAGAAGAAATTACCATGTCCGAGAAGCTACAACATGAGGCGATGGTTTTTGCCGATTTAGGTTGCTCTTCGGGTTCAAACACTTTGTCTTTCATCGCCCAAGTGCTTGACATCTTTGGTGAACATTGTCGGAGACTAGAAA**GAAAGCCACCGGAGATACAA**TTCTTCTTGAATGATCTCGTTGGAAATGACTTCAATTGTCTCTTTCATGCTTTAGAAGAATTTAATACAAAGAGGGGAGAATTATTTGTCCCATATTACGTTGTAGGAGTCCCAGGATCCTTTTACAAGAGACTTTTCCCTTGTGGATCTGTGAACCTCTTTCACTCTTCCTATTCTCTCCAGTGGCTCTCTCAGGTTCCTGATGGACTAAAAAATGATCAAGGTATTCCATTAAACAAACGAAATATCAATATTGCCGATGCAAGCCCATCTGAAGTTGTGAAAGCATATCAAAGACAATTTGAAAGAGATTTTTCAACGTTTCTCAAATATCGGCATGAAGAGCTAAGTA**TTGGAGGGGAAATGGTGTTA**ACATTCATAGGGAGAAAATCTAGACATCCAGTTGATAGCGAACTAAGTTGCCTGCATGGACTATTAGCAGATGCACTTAACTCAATGGTTTTGGAGGGAAAACTTGCACGAGATAAAGTGGACACCTTCGATATGCCAGTGTATGGACCTTCAATGGAGGAAGTGAAATCCATTATTTTTGCACAAGGTTTATTTGATCTCAAGCATGACCAAATGCTCAAGTATAGCTGGGATCCTTTTGTCGACTCATTGATTGATGCAACTCTTGACAATGTCCAACCAAGTGGGAGGAATATTGCTAAGTGTATACGGGCGGCGATAGAACCATTGATTTCACTTCAGTTTGGGGATGCCATAGTTGATGAAGTTTTTTCAAAATTTGCTGATAATATTTTTTGTTCTCTCCTGCCGGAGCAAGCTACATTTACCACACTTCTCATTGTGCTAAAGAAGACAATTTGAAGTTAAATAAAATCTATTGTGGAATTAAGCTCGGTAAGAGTGGGGCAAACATATATCTTTTAGTTATATTTTTATAATATATATACTTGGCACCCTCTATTAGACATAATCTTTGTAAATTTCAGTGGGGTAGATACCCACACTTGGGCCATATAGATCCGCTCATACTTGTGTACTAGCG

>gi|40642650|emb|AJ489277.1| Bixa orellana mRNA for lycopene cleavage oxygenase (lco gene)

ATGCAAGTCGAACCAACCAGGGGGATCGGCCTGGCGAACACAAGCTTGCAATTCTCCAACGGTCGACTCCACGCTCTATGTGAGTACGACCTCCCCTACGTTGTCCGCCTCTCCCCGGAAGACGGCGACATCTCAACCGTCGGACGCATCGAAAACAACGTCTCCACAAAAAGTACGACCGCCCACCCGAAGACGGATCCCGTCACCGGAGAGACGTTCAGCTTCAGCTACGGACCCATCCAGCCCTACGTCACGTACTCCCGTTATGACTGCGATGATAAGAAATCGGGCCCCGACGTGCCCATCTTCTCTTTCAAGGAACCGTCTTTCGTCCACGACTTCGCGATCACCGAACACTACGCCGTCTTCCCCGACATCCAGATCGTGATGAAGCCGGCCGAGATCGTTCGGGGGCGCCGCATGATCGGCCCGGACCTCGAGAAGGTCCCGAGGCTGGGGTTGCTTCCCCGGTACGCCACGTCGGACTCCGAGATGCGATGGTTCGACGTGCCAGGGTTCAACATGGTACACGTGGTGAACGCGTGGGAGGAGGAAGGCGGGGAGGTCGTGGTGATCGTGGCGCCCAACGTGAGCCCGATAGAGAACGCCATCGACCGGTTCGACCTCCTCCACGTGTCGGTGGAGATGGCGAGGATCGAGCTCAAGAGCGGGAGTGTGCCGCGGACGCTTCTCTCGGCGGAGAATCTGGATTTCGGGGTGATTCACCGGGGCTATTCGGGGAGGAAGAGCCGGTATGCTTACCTCGGAGTCGGGGATCCAATGCCGAAGATTCGCGGGGTGGTGAAGGTGGACTTCGAGTTGGCCGGGAGAGGGGAATGCGTGGTGGCGAGGAGGGAGTTCGGTGTGGGATGTTTCGGAGGAGAGCCGTTCTTTGTGCCGGCATCATCGAAGAAGTCCGGAGGCGAGGAAGACGATGGGTACGTAGTGAGTTACTTGCATGACGAGGGAAAGGGAGAGTCGAGTTTCGTGGTGATGGATGCGCGGTCGGCGCAGCTAGAGATCTTGGCGGAGGTGGTTCTGCCGCGGCGAGTGCCGTACGGGTTTCATGGCTTATTCGTTACGGACAAGGATCTGCTG**AACCAGGCATAAACAATCGG**CTAAGAAGATCTCTTTGTATAC
