# Supplementary material for: Next generation sequencing and de novo transcriptome analysis of Costus pictus D. Don, a non-model plant with potent anti-diabetic properties
Source: BMC Genomics. 2012 Nov 23;13:663. doi: 10.1186/1471-2164-13-663 (PMC3533581; doi:10.1186/1471-2164-13-663)
Supplement: Additional file 11 — Similarity search among other anti-diabetic plant resources. The file provides results of similarity search of the transcripts against GenBank nucleotide sequences from other anti-diabetic plants. [file 1471-2164-13-663-S11.doc]

**Similarity Search across other anti-diabetic plant resources**

The simiarity search results of the transcripts against nucleotide sequences from other anti-diabetic plants are displayed in the following table.

**Table: Similarity Search** Index

| **Species** | **Total Sequences in GenBank** | **Similar Transcripts** | **% of Similar Transcripts** |
| --- | --- | --- | --- |
| *Costus pictus* | 18 | 13 | 72.22 |
| *Costus speciosus* | 29 | 17 | 58.62 |
| *Syzygium cumini* | 15 | 5 | 33.33 |
| *Zingiber officinale* | 199 | 58 | 29.15 |
| *Vaccinium myrtillus* | 34 | 7 | 20.59 |
| *Panax quinquefolius* | 237 | 44 | 18.57 |
| *Rosmarinus officinalis* | 59 | 10 | 16.95 |
| *Momordica charantia* | 194 | 22 | 11.34 |
| *Gynostemma pentaphyllum* | 95 | 9 | 9.47 |
| *Trigonella foenum-graecum* | 47 | 1 | 2.13 |

The transcripts were compared against the available nucleotide sequences of anti-diabetic plants from Genbank database using megablast (Table: Similarity Search Index). Our attempts to compare the transcript profiles with other anti-diabetic plants indicated that *Costus pictus* is more closer to *Costus speciosus*. However, the sequences present in the Genbank database were far too less for a global comparison of the transcripts to find a similarity search index. The results are biased towards the presence and availability of housekeeping genes as well as species specific genes. For example, a species with low similarity might have an anti-diabetic activity, similar to that of *C. pictus*. However, the sequences encoding anti-diabetic properties might not have been submitted earlier in the database, which will lead to a false conclusion. Hence, it is difficult to conclude any result on similar modes of action from other plant species based on similarity from the above table alone.
