# Supplementary material for: Next generation sequencing and de novo transcriptome analysis of Costus pictus D. Don, a non-model plant with potent anti-diabetic properties
Source: BMC Genomics. 2012 Nov 23;13:663. doi: 10.1186/1471-2164-13-663 (PMC3533581; doi:10.1186/1471-2164-13-663)
Supplement: Additional file 12 — SNP filtering criteria. The file provides criteria used for filtering SNPs. [file 1471-2164-13-663-S12.doc]

**SNP Filtering Criteria**

A minimum read depth of 8, a minimum SNP quality of 20 and a minimum mapping quality of 20 were used to filter false positive SNPs. Because the reads were aligned against their respective assembled sequences, a small percentage of resulting homozygous SNPs were ruled out as mapping errors. Additionally, parameters from the following table were also considered for filtering false positive SNPs.

Table: The criteria for minimum number of reads showing variations ar particular read depths.

| **Number of reads aligned at a position (Read Depth)** | **Number of reads showing variation** |
| --- | --- |
| 08 to 20 | 4 |
| 21 to 30 | 6 |
| 31 to 40 | 8 |
| 41 to 50 | 10 |
| 51 to 60 | 12 |
| 61 to 70 | 14 |
| 71 to 80 | 16 |
| 81 to 90 | 18 |
| >91 | 20 |
